# Antibodies against High Mobility Group Box protein-1 (HMGB1) versus other anti-nuclear antibody fine-specificities and disease activity in systemic lupus erythematosus

**DOI:** 10.1186/s13075-015-0856-2

**Published:** 2015-11-23

**Authors:** Lina Wirestam, Hanna Schierbeck, Thomas Skogh, Iva Gunnarsson, Lars Ottosson, Helena Erlandsson-Harris, Jonas Wetterö, Christopher Sjöwall

**Affiliations:** AIR, Department of Clinical and Experimental Medicine, Linköping University, SE-581 85 Linköping, Sweden; Unit of Pediatric Rheumatology, Department of Women’s and Children’s Health, Karolinska Institutet, Karolinska University Hospital, Solna, Karolinska Institutet, Stockholm, Sweden; Rheumatology Unit, Department of Medicine, Karolinska Institutet, Karolinska University Hospital, Stockholm, Sweden

**Keywords:** HMGB1, Autoantibodies, SLE, Antinuclear antibodies, Inflammation, Clinical phenotype, Complement proteins

## Abstract

**Introduction:**

The non-histone nuclear protein high mobility group box protein-1 (HMGB1) is typically associated with nucleosomes, but may shuttle between the nucleus and the cytoplasm, and under some conditions also be released extracellularly and participate in systemic inflammation. Monoclonal HMGB1-targeting antibodies can ameliorate murine polyarthritis and lupus-like disease. Interestingly, autoantibodies against HMGB1 have also been described in patients with systemic lupus erythematosus (SLE), but their clinical implications remain elusive. The main aims of this study were to detect serum anti-HMGB1 antibodies in patients with SLE and relate them to other types of antinuclear antibodies (ANA), and to disease activity.

**Methods:**

188 Swedish SLE patients meeting the 1982 American College of Rheumatology classification criteria and/or the 2012 Systemic Lupus International Collaborating Clinics classification criteria participated in the study. Anti-HMGB1 antibody levels were analysed in patient and control (n = 112) sera by an in-house ELISA using recombinant histidine-tagged HMGB1. SLE sera were also analysed for ANA by immunofluorescence (IF) microscopy (IF-ANA) using fixed HEp-2 cells, and by a line-blot assay for antigen fine-specificities. To quantify antibodies to double-stranded DNA, a fluoroenzyme-immunoassay was employed.

**Results:**

At inclusion, 23 % of the SLE patients were anti-HMGB1 antibody positive compared to 5 % of the controls. Anti-HMGB1 antibodies occurred in 49 % of the IF-ANA positive SLE patients, and in 34 % of IF-ANA negative cases (p = 0.004). Levels of anti-HMGB1 antibodies correlated with anti-dsDNA antibody levels (r = 0.49; p < 0.001). Significant, but less pronounced correlations were found regarding anti-HMGB1 and SLE disease activity index (SLEDAI-2K: r = 0.15; p = 0.04), classical complement function (r = -0.24; p = 0.002) and complement protein C4 (r = -0.23; p = 0.002). Average anti-HMGB1 antibody levels were significantly higher among patients with homogenous ± other IF-ANA staining patterns (median 180 AU) compared to IF-ANA negative cases (median 83 AU) (p = 0.004). Rabbit anti-HMGB1 antibodies gave rise to cytoplasmic, but not nuclear, staining of HEp-2 cells.

**Conclusions:**

We confirm that anti-HMGB1 antibodies are common in SLE and correlate with disease activity variables. Although anti-HMGB1 antibodies measured by ELISA often coincide with nuclear IF-ANA staining, our results indicate that anti-HMGB1 antibodies do not give rise to nuclear staining of the predominantly used commercial HEp-2 cell slides.

**Electronic supplementary material:**

The online version of this article (doi:10.1186/s13075-015-0856-2) contains supplementary material, which is available to authorized users.

## Introduction

Abnormally high serum levels of antinuclear antibodies (ANA) assessed by indirect immunofluorescence (IF) microscopy (IF-ANA) is one of the 11 classification criteria for systemic lupus erythematosus (SLE) according to the American College of Rheumatology (ACR) 1982 and the suggested update 1997 [1, 2]. Applying a cut-off level >95^th^ percentile among healthy female blood donors, IF-ANA occurs in the large majority (98–99 %) of SLE patients at diagnosis [3], although the point prevalence among established cases is considerably lower [4–6]. Depending on the many different nuclear target antigens for ANA, different IF-staining patterns can be seen. Thus, antibodies against double-stranded (ds) DNA, histones and DNA-histone complexes typically produce a homogeneous nuclear staining pattern on non-dividing cells, and staining of the condensed chromatin-associated antigens in mitotic cells. In contrast, ANA specific for extrachromosomal antigens can be identified as a speckled nuclear staining pattern in non-dividing cells, and diffuse extra-chromosomal staining of dividing cells. In addition, other IF-ANA staining patterns can be distinguished on HEp-2 cells (e.g., centromeric and nucleolar patterns) and indicate other antigen specificities and clinical characteristics [7, 8].

Increased apoptosis and impaired clearance of apoptotic material results in raised levels of circulating autoantigens and increased exposure of these antigens to the adaptive immune system. This may underlie the excessive production of ANA in SLE, and the formation of circulating and tissue-bound immune complexes (ICs), which probably contribute to SLE pathogenesis [9–11]. Thus, uncontrolled autoimmune responses, abnormal formation of autoantibodies/ICs, and increased extrahepatic IC deposition may promote inflammation and result in a large variety of clinical manifestations ranging from skin rash and arthritis to cytopenia, nephritis, and neurological symptoms [3].

High mobility group box-1 protein (HMGB1) was originally discovered as a 25 kDa DNA-binding protein that participates in many nuclear functions, e.g., regulation of gene transcription, chromatin replication and DNA repair [12]. Triggered by trauma, infection and other pro-inflammatory stimuli, HMGB1 can also be released extracellularly and act as a pro-inflammatory mediator, e.g., inducing monocyte synthesis of pro-inflammatory cytokines and chemokines [13–16]. The pro-inflammatory functions of HMGB1 are determined by the configurations of the oxidative states on the three cysteine residues, C_23_, C_45_ and C_106_ [17]. HMGB1 can also instantly leak extracellularly from dying cells due to ruptured plasma membranes [18]. During silent apoptotic cell death, HMGB1 is normally not released, provided that the apoptotic material is properly engulfed and degraded by phagocytic cells. However, due to the insufficient clearance of apoptotic debris in SLE, secondary necrosis will occur and significant amounts of DNA-attached HMGB1 will be released [19]. Such DNA-bound HMGB1 is not cytokine-inducing due to irreversible oxidation of the critical cysteine at position 106 that is essential for HMGB1-mediated cytokine induction [20]. However, HMGB1 attached to DNA or by itself is highly immunogenic as a nuclear autoantigen irrespective of its redox state, and stimulates the production of autoantibodies [21, 22].

In SLE, HMGB1 has been shown to potentiate the production of ANA recognising nucleosomes and dsDNA [21, 22]. HMGB1 bound to DNA-containing ICs has also been shown to induce interferon-alpha (IFN-α) production by plasmacytoid dendritic cells, and to activate autoreactive B cells [23]. Serum levels of HMGB1 are elevated in SLE patients and have been found to correlate positively with disease activity [24–27], and inversely with levels of complement proteins C3 and C4 [26, 28]. HMGB1 may also serve as an autoantigen, leading to the production of anti-HMGB1 antibodies [26, 29–32]. Anti-HMGB1/HMGB2 antibodies were first described as a type of “perinuclear” anti-neutrophil cytoplasm antibody (ANCA) in ulcerative colitis [29]. Circulating anti-HMGB1 antibodies have also been reported in SLE [26, 30–32] where the antibody levels appear to correlate with the SLE disease activity index (SLEDAI) and coincide with renal involvement [26]. However, the possible biological/clinical importance of anti-HMGB1 antibodies remains to be clarified.

The present study was undertaken to further elucidate the occurrence, and clinical and serological correlates of anti-HMGB1 antibodies in patients with SLE. Due to the typical nuclear localization of HMGB1, anti-HMGB1 antibodies were furthermore evaluated as a potential source of IF-ANA.

## Methods

### Patients and control subjects

Patients diagnosed with SLE (n = 188; 167 women, 21 men; mean age 49.1 years; age range 18–88 years) were included in the study. All patients took part in the prospective, structured follow-up program “KLURING” (Swedish acronym for Clinical LUpus Register In Northeastern Gothia) [7, 33, 34] at the rheumatology outpatient clinic, Linköping University Hospital, Sweden. Of the 188 patients, 163 (87 %) met at least 4 of the 1982 American College of Rheumatology classification criteria (ACR-82). Another 25 patients (13 %) solely fulfilled the 2012 Systemic Lupus International Collaborating Clinics (SLICC) classification criteria [35], whereas 158 patients (84 %) met both ACR-82 and SLICC-12 criteria. The patients were recruited consecutively. Most were prevalent cases (165 patients, 88 %), but 23 patients (12 %) had recent-onset disease at the time of sampling. The mean disease duration was 11 years (range 0–45 years). The SLE disease activity index 2000 (SLEDAI-2K) was recorded at each visit [36] and the SLICC/ACR damage index was registered prospectively after inclusion in KLURING [37]. Further characteristics of the patients are summarized in Table 1.

**Table 1:** Characteristics of the SLE patients (n = 188) in relation to the presence of anti-HMGB1

	Mean (range) or %			*P* value^a^
	All patients n = 188	Anti-HMGB1 positive n = 43	Anti-HMGB1 negative n = 145	
Age, years	49.1 (18–88)	44.9 (19–80)	50.4 (18–88)	ns
Females	88.8 %	88.4 %	89.0 %	ns
Disease duration, years	10.9 (0–45)	10.9 (0–39)	10.9 (0–45)	ns
Prednisolone dosage, mg/day	5.1 (0–60)	8.3 (0–60)	4.2 (0–25)	ns
SLICC/ACR damage index, score	1.1 (0–8)	1.2 (0–8)	1.1 (0–8)	ns
SLEDAI-2K, score	2.5 (0–24)	3.1 (0–24)	2.3 (0–16)	ns
PGA, score	0.4 (0–4)	0.6 (0–4)	0.4 (0–3)	ns
Patients meeting SLICC-12, n (%)	183 (97.3)	42 (97.7)	141 (97.2)	ns
Fulfilled ACR-82 criteria, n	4.8 (3–9)	5.0 (3–9)	4.7 (3–8)	ns
ACR-82 criteria, n (%)				
1. Malar rash	87 (46.3)	20 (46.5)	67 (46.2)	ns
2. Discoid rash	32 (17)	7 (16.3)	25 (17.2)	ns
3. Photosensitivity	103 (54.8)	23 (53.5)	80 (55.2)	ns
4. Oral ulcers	21 (11.2)	6 (14)	15 (10.3)	ns
5. Arthritis	144 (76.6)	35 (81.4)	109 (75.2)	ns
6. Serositis	72 (38.3)	19 (44.2)	53 (36.6)	ns
7. Renal disorder	43 (22.9)	10 (23.3)	33 (22.8)	ns
8. Neurologic disorder	11 (5.9)	3 (7)	8 (5.5)	ns
9. Hematologic disorder	106 (56.4)	20 (46.5)	86 (59.3)	ns
10. Immunologic disorder	92 (48.9)	29 (67.4)	63 (43.4)	0.009
11. IF-ANA	185 (98.4)	42 (97.7)	143 (98.6)	ns

^a^Mann–Whitney *U* test and Chi^2^ test, respectively. *HMGB1* high mobility group box protein-1, *SLEDAI-2K* systemic lupus erythematosus disease activity index 2000, *SLICC* Systemic Lupus International Collaborating Clinics, *ACR* American College of Rheumatology, *PGA* physician’s global assessment, *IF-ANA* immunofluorescence antinuclear antibodies, *ns* not significant

Randomly selected age- and sex-matched individuals from the general population (n = 112; 102 women, 10 men; mean age 47 years; range 19–84 years), none of whom had a diagnosis of SLE, were recruited from the Swedish population register, and served as controls for the anti-HMGB1 antibody analyses.

Peripheral venous blood was drawn from each individual at baseline. Serum samples were prepared and stored at −70 °C until analyzed. Eighteen patients, all meeting the ACR-82 criteria, were selected for consecutive analyses (2–13 visits per patient), due to fluctuations in disease activity over time (i.e., SLEDAI-2K peak score of at least 4 points).

### Production of recombinant HMGB1

Recombinant rat histidine-tagged HMGB1 cDNA was cloned into pET28a vector (Clontech, Mountain View, CA, USA) as previously published [38] and transformed into BL21 (DE3) strain (Stratagene, Santa Clara, CA, USA). The protein, with 99 % homology to human HMGB1, was purified with Ni Sepharose High Performance affinity media (GE Healthcare, Chalfont St. Giles, UK) according to the protocol supplied by the manufacturer, followed by gel filtration on a Superdex 75 column (GE Healthcare) with phosphate-buffered saline (PBS), pH 7.4, as running buffer. Endotoxin was removed by addition of 1 % Triton X-114 and incubation at 4 °C for 30 minutes, followed by incubation in a 37 °C water bath for 10 minutes, and subsequently centrifugation at 18,300 g/25 °C for 10 minutes. This procedure was repeated once and yielded endotoxin levels below 0.003 EU/μg protein, according to the *Limulus* amoebocyte lysate assay (analyzed by the clinical laboratory at Karolinska University Hospital, Stockholm, Sweden). The protein preparation was also free from DNA as evaluated by agarose gel electrophoresis and staining for DNA with GelRed (Biotium, Hayward, CA, USA).

### Anti-HMGB1 autoantibodies

Autoantibodies against HMGB1 were measured by an in-house enzyme-linked immunosorbent assay (ELISA). Briefly, Nunc maxisorp 96-well plates (Thermo Fisher Scientific, Uppsala, Sweden) were coated with recombinant rat histidine-tagged HMGB1 (10 μg/ml in 50 mM carbonate buffer, pH 9.6) overnight at 4 °C. The well surfaces were blocked by incubation with 5 % non-fat dry milk powder (Bio-Rad, Hercules, CA, USA) in PBS for 30 minutes. Serum samples were diluted 1:500 in PBS/0.05 % Tween/1 % milk powder and a 7-point standard curve with pooled positive SLE sera were prepared (starting at dilution 1:500 (=1600 arbitrary units (AU)) followed by serial two-fold dilutions). Samples and standards were incubated in the wells for 2 hours at room temperature. Secondary horseradish peroxidase-conjugated rabbit anti-human IgG antibody (Dako, Glostrup, Denmark) was diluted 1:2000 in PBS/0.05 % Tween/1 % milk powder, added to the plate and incubated at room temperature for 2 hours. Plates were developed with tetramethylbenzidine substrate (Sigma-Aldrich, St. Louis, MO, USA). The reaction was stopped by adding 2M H_2_SO_4_. AU were calculated by normalization against a standard pool of IgG anti-HMGB1-positive serum samples from 11 different SLE patients. The cutoff value of 300 AU was calculated based on the mean value of anti-HMGB1 + two standard deviations among the 112 referents.

### Indirect immunofluorescence microscopy for ANA patterns and HMGB1 localisation

SLE sera diluted 1:200 were also analyzed for IF-ANA using multispot slides with fixed HEp-2 cells (ImmunoConcepts, Sacramento, CA, USA). At this cut-off limit <5 % of healthy female blood donors test ANA-positive [4]. The HEp-2 cell slides were incubated with PBS-diluted sera for 30 minutes, washed with PBS for 10 minutes, and incubated with fluorescein-isothiocyanate (FITC)-conjugated γ-chain-specific rabbit polyclonal anti-human IgG (DAKO). After incubation and washing, the microscope slides were mounted with Fluorescence Mounting Medium (DAKO) and cover slips. The microscope prerequisites have been specified previously [4]. Based on the immuno-morphological staining pattern, samples were categorized into three groups: 1) ANA-negative, 2) homogenous ANA ± other ANA patterns, and 3) other ANA patterns: speckled, centromeric, nucleolar or multiple nuclear dots.

For immunomorphological localization of HMGB1, fixed HEp-2 cells (see above), and non-fixed 5-μm cryostat sections of rat liver, respectively, were incubated for 30 minutes with polyclonal rabbit anti-HMGB1 (Abcam, Cambridge, UK; dilution 1:100 in PBS). After PBS washing, the slides were incubated for 30 minutes with FITC-conjugated polyclonal goat anti-rabbit IgG-Fc diluted 1:50 (Abcam).

### ANA fine specificity

ANA fine specificities were analyzed using a line blot kit (ANA Profile 5, EUROIMMUN, Lübeck, Germany). The assay was run according to the manufacturer’s instructions on an automated EUROBlotmaster (EUROIMMUN) instrument. Briefly, the antigen-coated immunoblot strips were incubated for 30 minutes with serum samples diluted 1:101 with PBS. After washing, alkaline phosphatase-labeled anti-human IgG, diluted 1:10, was added and incubated for 30 minutes. After washing, substrate solution (nitrobluetetrazoliumchlorid/5-bromo-4-chloro-3-indolylphosphate) was added for 10 minutes. All incubations were performed at room temperature. The enzymatic reaction was stopped by washing the strips with distilled water and the blot intensities were automatically semi-quantified by densitometry with the EUROLineScan (EUROIMMUN). Cut-off levels for positive tests were set to a signal intensity of ≥11 arbitrary units according to the manufacturer’s suggestion. Apart from HMGB1, the three isolated target antigens of interest in this study were: 1) histone-1 stripped nucleosomes (Nu2) derived from calf thymus, 2) a mixture of histone H1 and H2b purified from calf thymus, and 3) dsDNA (isolated from salmon testes).

### Fluoroenzyme-immunoassay for quantification of anti-dsDNA antibodies

Anti-dsDNA detections were performed using the Phadia250 instrument (EliA™ dsDNA; Thermo Fisher Scientific, Uppsala, Sweden) as described elsewhere [39]. Briefly, serum samples were added to antigen-coated wells, where they were diluted 1:10. After incubation and washing, monoclonal γ-chain specific anti-human IgG conjugated with β-galactosidase was added. Development solution (0.01 % 4-methylumbelliferyl-β-D-galactoside) was then applied, and the reaction subsequently terminated by adding 4 % sodium carbonate. An autoantibody concentration ≥16 IU/ml was considered positive according to the manufacturer’s suggestion. Samples above the assay range (≥379 IU/ml) were given a value of 450 IU/mL in statistical analyses.

### Other laboratory analyses

At all patient visits, routine laboratory analyses were performed at Linköping University Hospital, apart from classical complement function in fresh frozen plasma samples, which was analyzed at Uppsala University Hospital.

### Ethics

Oral and written informed consent was obtained from all SLE subjects. The study protocol was approved by the Regional Ethics Review Board in Linköping (Dnr: M75-08/2008). The Regional Ethics Review Board in Stockholm approved the part of the study involving control subjects.

### Statistical analyses

The Mann–Whitney *U* test was used to evaluate differences in anti-HMGB1 levels between patients and controls. In order to distinguish differences in anti-HMGB1 levels between ANA-groups, Kruskal–Wallis with Dunn’s multiple comparison post-hoc test was used. Spearman’s correlation was used to determine the association between anti-HMGB1 and disease variables. Two-tailed *P* values <0.05 were considered significant. Statistical analyses were performed with SPSS Statistics 22 (IBM, Armonk, NY, USA) or GraphPad Prism 5, version 5.04 (GraphPad Software, La Jolla, CA, USA).

## Results

### IF-ANA and anti-HMGB1 antibodies

**Fig. 1:**
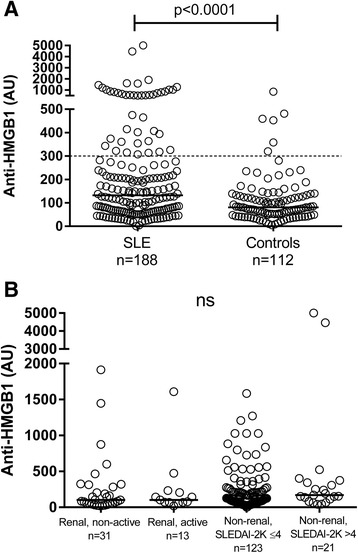
Serum anti-high mobility box protein-1 (*HMGB1*) antibody levels determined by ELISA. **a** The average level of anti-HMGB1 was significantly higher in the systemic lupus erythematosus (*SLE*) patients (median 132.5 arbitrary units (*AU*)) compared to the healthy controls (median 81 AU). *Dashed line* indicates the cut-off level for a positive test. *Solid lines* represent median values. Note the axis break. **b** Serum levels in SLE patients categorised into renal non-active, renal active (see text for definitions), non-renal active with systemic lupus erythematosus disease activity index (SLEDAI)-2K ≤4, and non-renal active with SLEDAI-2K >4

At inclusion, 23 % of the SLE patients were anti-HMGB1 antibody-positive compared to 5 % of the controls (Fig. 1a). The average level of anti-HMGB1 antibodies was significantly higher (*P* <0.0001) among the SLE patients (median 132.5 AU) compared to the healthy controls (median 81 AU) (Fig. 1a). To evaluate the importance of anti-HMGB1 antibodies in renal SLE, we compared anti-HMGB1 antibody levels in patients meeting the ACR-82 classification criterion for renal disorder and patients who did not fulfil this criterion. Furthermore, patients were categorized by disease activity in the renal domain (i.e., presence of urinary casts, hematuria, proteinuria and leukocyturia), and into active (SLEDAI-2K >4) and non-active (SLEDAI-2K ≤4). No significant differences were found between the groups (Fig. 1b).

Levels of anti-HMGB1 antibodies were also analyzed in the consecutive serum samples from 18 patients: 11 (61 %) of these tested positive on at least one occasion. Anti-HMGB1 levels were compared at the highest and lowest disease activity (defined by SLEDAI-2K), but no significant differences were found (Additional file 1). See Additional file 2 for longitudinal data on each of the 11 individuals who were ever anti-HMGB1-positive.

As shown in Table 1, 98.4 % of the 188 SLE patients included in this study had ever been IF-ANA-positive, 97.7 % among anti-HMGB1-positive patients and 98.6 % among anti-HMGB1-negative patients. On further comparison of anti-HMGB1-positive and anti-HMGB1-negative SLE patients there were no differences in baseline data on age, sex, disease duration, prednisolone medication, disease activity, or disease phenotype based on the ACR-82 classification criteria (Table 1).

**Fig. 2:**
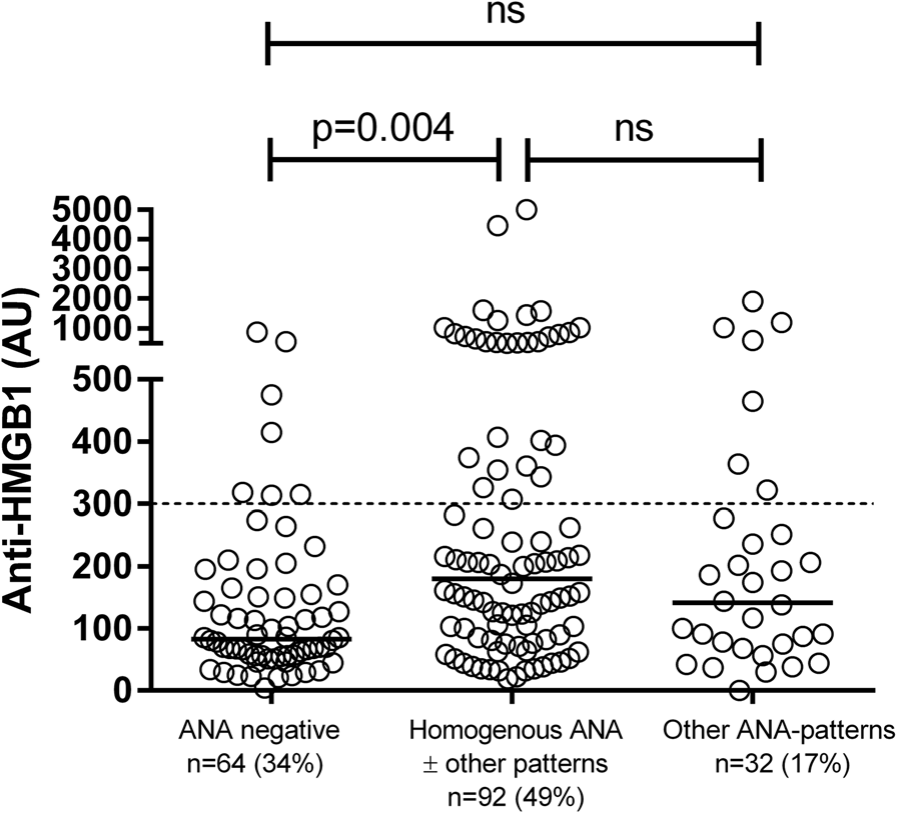
Indirect immunofluorescence (IF) microscopy for antinuclear antibodies (*ANA*) and anti-high mobility box protein-1 (*HMGB1*) antibodies. Serum levels determined by ELISA of anti-HMGB1 among systemic lupus erythematosus patients grouped according to immunofluorescence patterns of ANA. There were 34 % ANA-negative and 66 % ANA-positive patients. IF-ANA-positive patients with a homogenous nuclear staining pattern had significantly higher levels of anti-HMGB1 antibodies compared to the IF-ANA negative group. *Dashed line* indicates cut-off level for a positive anti-HMGB1 test; *solid lines* represent median anti-HMGB1 levels. Note the axis break. *AU* arbitrary units

The present serologic point prevalence results, however, revealed that only 124 (66 %) of the SLE cases were IF-ANA-positive. Among the ANA-positive patients, 74 % of the sera produced a homogenous (chromosomal) IF-ANA staining pattern (with or without other patterns), and 26 % thus had other (extra-chromosomal) IF-ANA staining patterns. The IF-ANA-positive patients with a homogenous nuclear staining pattern had significantly (*P* = 0.004) higher levels of anti-HMGB1 antibodies (median 180 AU) compared to the IF-ANA-negative group (median 83 AU), whereas the ANA-positive patients with other nuclear (non-homogenous) staining patterns did not differ in anti-HMGB1 status from the ANA-positive patient group (Fig. 2).

### Anti-HMGB1 antibodies versus disease activity measures

Levels of anti-HMGB1 antibodies correlated positively with anti-dsDNA antibody levels as analyzed by EliA™ (*r* = 0.49; *P* <0.001). Less pronounced correlations were found regarding disease activity (SLEDAI-2K; *r* = 0.15; *P* = 0.04), classical complement function (*r* = –0.24; *P* = 0.002) and plasma levels of C4 (*r* = –0.23; *P* = 0.002). There was no significant association with disease phenotypes (Table 1) or organ damage (SLICC/ACR damage index).

### Anti-HMGB1-positive patients with IF-ANA reactivity to chromatin-associated antigens

**Fig. 3:**
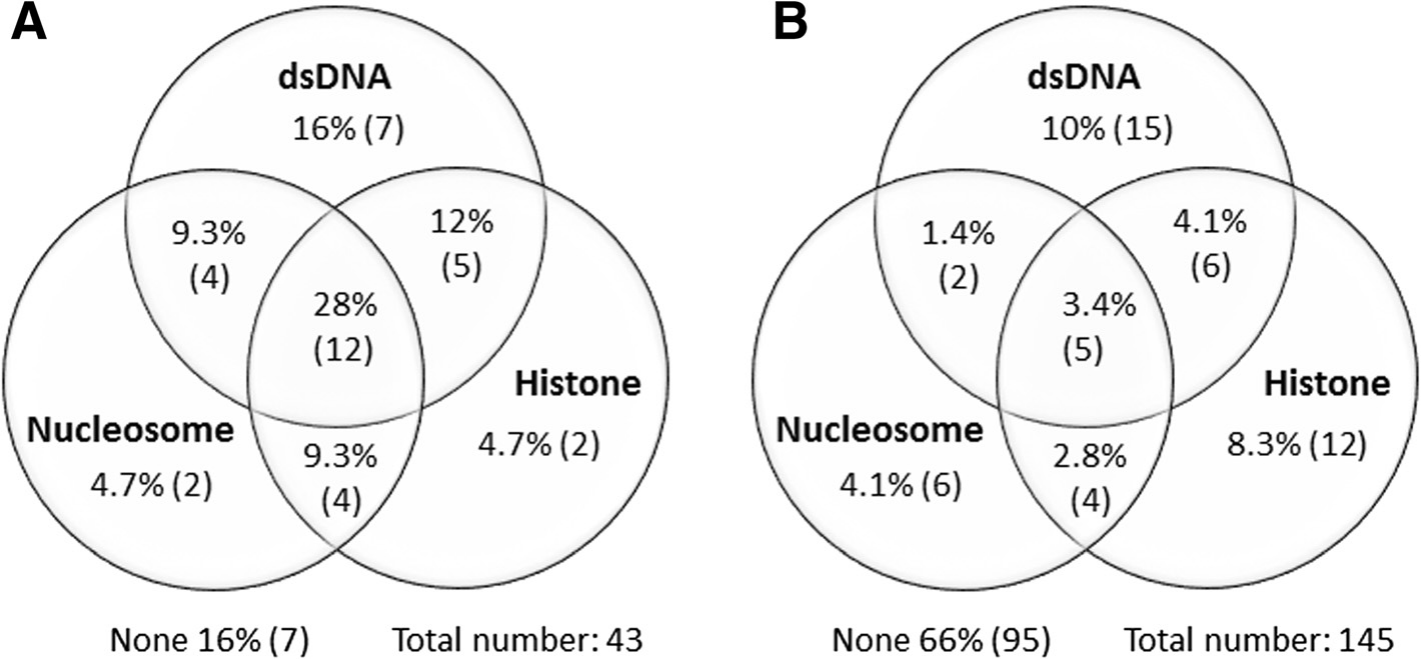
Antinuclear antibodies (ANA) specificity in anti-high mobility box protein-1 (HMGB1)-positive patients. Overlap of ANA specificity in anti-HMGB1 antibody-positive systemic lupus erythematosus (SLE) patients (**a**) and anti-HMGB1 antibody-negative SLE patients (**b**), measured with a line blot technique detecting anti-dsDNA, anti-histone and anti-nucleosome antibodies

As illustrated in Fig. 3a, among the 43 SLE patients testing positive for anti-HMGB1 antibodies, 7 (16 %) also had anti-dsDNA antibodies analyzed by the line blot assay, 2 (4.7 %) had anti-nucleosome Nu2 antibodies (line blot), and 2 (4.7 %) had anti-histone reactivity (line blot): 12 of the 43 anti-HMGB1-positive patients (28 %) had antibodies reactive to dsDNA and to nucleosome Nu2 and histones.

In contrast to the anti-HMGB1-positive patients, only 3.4 % of the anti-HMGB1-negative SLE patients were triple-positive for antibodies against dsDNA, Nu2, and histones, while 66 % were negative for all of these ANA specificities (Fig. 3b).

### Cellular localization of HMGB1

**Fig. 4:**
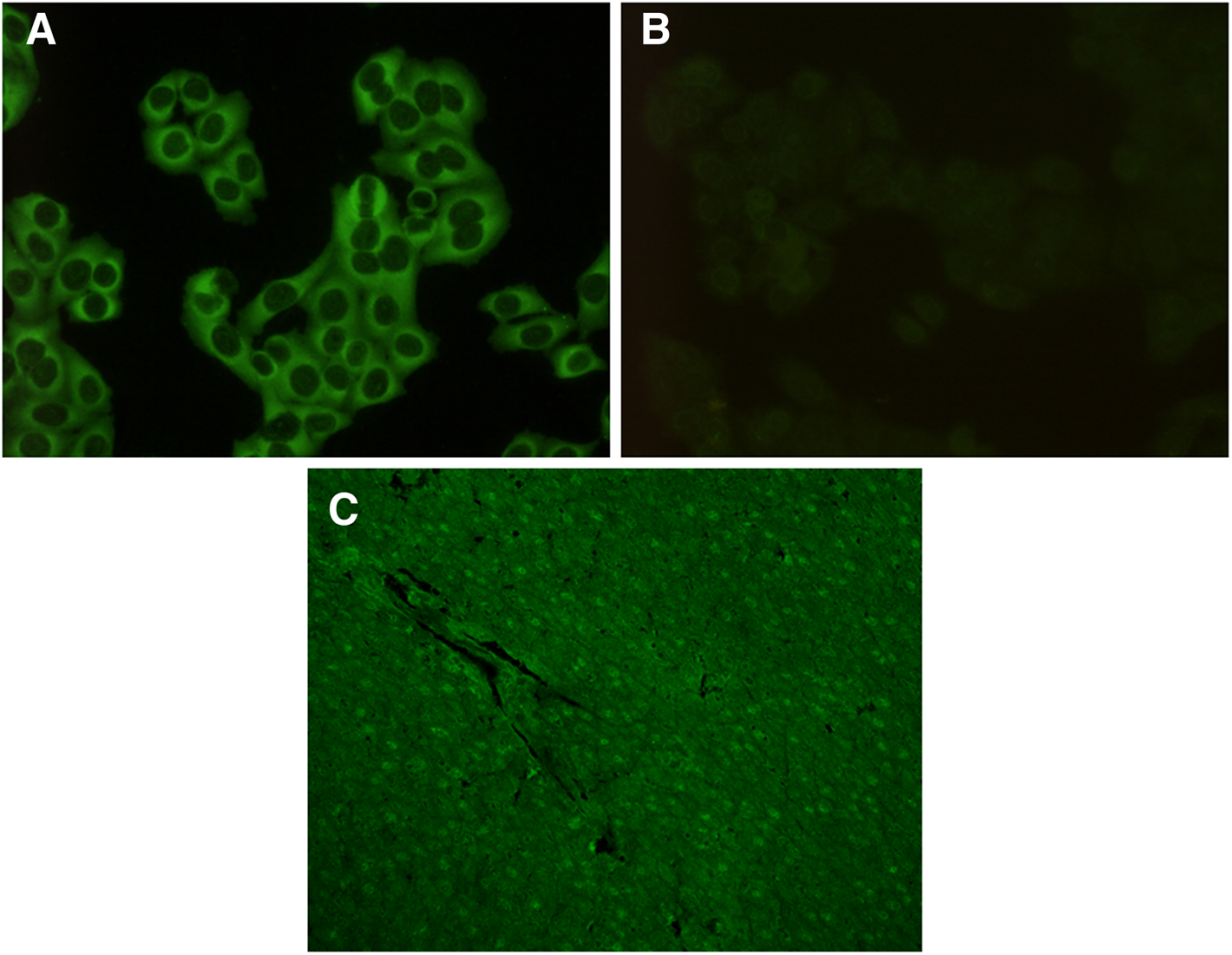
Cellular localization of high mobility box protein-1 (HMGB1). Indirect immunofluorescence microscopy images. **a** Cytoplasmic/extra-chromosomal HEp-2 cell immunofluorescence after incubation with rabbit IgG anti-HMGB1 followed by FITC-conjugated anti-rabbit IgG. **b** Negative control HEp-2 cells incubated with fluorescein-isothiocyanate (FITC)-conjugated anti-rabbit IgG alone. **c** Hepatocyte nuclear fluorescence after incubation of rat liver cryostat section with rabbit anti-HMGB1 and FITC anti-rabbit IgG

We could neither identify HMGB1 in nuclei of non-dividing HEp-2 cells, nor in the chromatin of dividing cells. Instead, a diffuse cytoplasmic staining was seen here, but not in the control slides incubated with detection antibody alone (Fig. 4a–b). However, in unfixed rat liver cryostat sections a faint IF-ANA reaction with a homogenous nuclear staining pattern was observed in the hepatocytes indicating nuclear localization of HMGB1 (Fig. 4c).

## Discussion

Anti-HMGB1 antibodies have previously been reported in SLE patients [26, 31, 32] and there are also a few reports on elevated HMGB1 levels in SLE [24–26, 40]. However, the clinical value and possible role of anti-HMGB1 antibodies in the pathogenesis of SLE remains elusive and further studies on this matter are warranted. Apart from SLE, the presence of anti-HMGB1 antibodies has been reported in other chronic inflammatory diseases [29, 30, 41–43]. The present study was undertaken to determine the levels of anti-HMGB1 antibodies in sera from SLE patients in relation to other disease variables.

By utilizing a novel ELISA, we confirmed that anti-HMGB1 antibodies are significantly increased in SLE as compared to healthy controls. Among the 188 SLE patients included in the present study, we found that IgG anti-HMGB1 antibodies were present in about 1/4 of the cases. Circulating levels of anti-HMGB1 antibodies had a fair degree of correlation with anti-dsDNA antibody levels and less pronounced, but statistically significant, correlation with disease activity markers such as classical complement activation, reduced levels of complement protein C4 and with disease activity index SLEDAI-2K. However, we did not observe any significant correlation between fluctuations of anti-HMGB1 levels and SLE disease activity in the 18 patients for whom we had prospective data (see Additional file 2). The association between anti-HMGB1 antibodies and renal involvement was not as obvious in the present study compared to the observation by Abdulahad et al. [26]. The reason for this discrepancy may be due to both differences in study population (ethnicity, SLE phenotypes and disease activity) and in methodology. The amino acid sequence of the recombinant HMGB1 used in this study originates from rat. The sequence homology between rat and human HMGB1 is 99 % and the differences lie within the highly acidic C-terminal tail. Aspartic acids and glutamic acids have been interchanged in three positions, however no functional differences nor any impact on antibody epitopes have been reported in the literature [31].

A positive IF-ANA test is a hallmark of SLE [44]. HMGB1 is a non-histone nucleosomal protein (although it can shuttle to the cytoplasm and become released extracellularly). Therefore, it is not farfetched to assume that anti-HMGB1 antibodies should give rise to a homogenous ANA staining pattern in conventional IF-ANA tests on HEp-2 cells, i.e., the predominating clinical IF-ANA test worldwide. Indeed, we found that a positive anti-HMGB1 antibody test by ELISA was predominantly associated with a homogenous IF-ANA (with or without other ANA patterns). Surprisingly, however, we found that incubation with polyclonal rabbit anti-HMGB1 antibodies did not generate nuclear staining on HEp-2 cells, but rather a diffuse cytoplasmic staining pattern. As the HEp-2 cells are derived from a human adenocarcinoma, and as the location of HMGB1 may predominate in the cytoplasm of malignant cells [45, 46], this could be a plausible explanation for the cytoplasmic staining of HMGB1 in HEp-2 cells. Another possible explanation for the cytoplasmic location of HMGB1 could be a fixation artifact with redistribution of nuclear HMGB1 to the cytoplasm. The importance of fixatives has been highlighted previously, e.g., as regards distribution of cellular (membrane-bound, cytoplasmic, nuclear) antigens [47–49], including HMGB1 [30]. Whatever the reason for divergent staining patterns yielded by anti-HMGB1 antibodies when applied to fixed HEp-2 cells, as compared to unfixed rat liver sections, it is highly unlikely that serum anti-HMGB1 antibodies will be identified as typical positive IF-ANA when the commercially available HEp-2 cell substrates are used in clinical laboratory routine setups for IF-ANA diagnostics. The seemingly low point prevalence (66 %) of IF-ANA-positive patients in our cohort of prevalent cases may appear bothering, but is well in line with what we and others have shown applying a cut-off level for positive IF-ANA ≥95^th^ percentile among healthy female controls [4–6, 50].

The possible biological roles of anti-HMGB1 antibodies have been considered in relation to other inflammatory disorders. For instance, the presence of autoantibodies to HMGB1 in sepsis has been shown to be associated with increased survival among critically ill patients, thus indicating that the induction of autoantibodies can be beneficial in infectious diseases [51]. Studies in animal models of arthritis and lupus have shown that treatment with anti-HMGB1 antibodies can strikingly attenuate disease by blocking the pro-inflammatory function of HMGB1 [52–54]. Administration of a neutralising monoclonal anti-HMGB1 antibody to the lupus-prone BXSB mice attenuates proteinuria, glomerulonephritis, circulating anti-dsDNA, immune complex deposition and levels of cytokines in serum [53].

HMGB1 that is passively released during secondary necrosis is not cytokine-inducing due to irreversible oxidation of the three cysteines that are required for HMGB1 function as a cytokine inducer [20]. Actively secreted HMGB1 is acetylated, in contrast to the passively released HMGB1 [55]. In order to fully elucidate the role of HMGB1 (and anti-HMGB1 antibodies) in SLE, the molecular isoform of HMGB1 (and antibody fine-specificities) must be determined. However, analyzing the isoform of extracellular HMGB1 can today only be measured by analytical tandem mass spectrometry [56], which is a time-consuming approach not applicable for studying large patient cohorts and was beyond the scope of the present study. To study molecular isoforms could potentially identify the source of extracellular HMGB1 that will further clarify the role of HMGB1 in SLE and also the generation of anti-HMGB1 antibodies.

Although anti-HMGB1 antibodies occur in patients with different inflammatory disorders, it does not necessarily imply, nor exclude, that they are pathogenic. Based on the therapeutic studies described above, anti-HMGB1 autoantibodies may hypothetically even be beneficial in some instances. Obviously, the question of whether anti-HMGB1 antibodies have protective/neutralising or pathogenic roles in SLE needs further investigations.

## Conclusion

We confirm that anti-HMGB1 antibodies occur in SLE and correlate with disease activity variables. Although anti-HMGB1 antibodies measured by ELISA often coincide with nuclear staining, our results indicate that anti-HMGB1 antibodies do not give rise to nuclear staining of the widely used HEp-2 cell IF-ANA microscopy slides.

## Additional files

Additional file 1:
**Figure shows anti-high mobility group box protein-1 (** ***HMGB1*** **) antibody levels at highest and lowest disease activity.** Highest and lowest disease activity was defined by peak systemic lupus erythematosus disease activity index (*SLEDAI*)-2K score and remission in the 18 patients selected for consecutive analysis. *Dashed line* indicates cut-off level for positive test. (TIF 150 kb) Additional file 2:
**Figure shows individual SLE manifestations and longitudinal variations of anti-high mobility group box protein-1 (** ***HMGB1*** **), anti-dsDNA, classical complement function and systemic lupus erythematosus disease activity index (** ***SLEDAI*** **)-2K.** The graphs illustrate individual variations in anti-HMGB1 (cut-off ≥300 U/ml), anti-dsDNA (EliA; cut-off ≥16 IU/ml), classical complement function (reference interval 80 - 120 %) and SLEDAI-2K levels over time for the 11 individuals who were ever anti-HMGB1 positive (*B-L*), who were followed consecutively. The first graph (A) is an example graph describing titles and lines of the other graphs. Observe that axes and scales are different in the graphs. (TIF 1063 kb)

## Abbreviations

ACR: American College of Rheumatology
ANA: antinuclear antibodies
AU: arbitrary units
ELISA: enzyme-linked immunosorbent assay
FITC: fluorescein-isothiocyanate
HMGB1: high mobility group box protein-1
IC: immune complex
IF: immunofluorescence
kDa: kiloDalton
KLURING: Clinical Lupus Register in Northeastern Gothia
PBS: phosphate-buffered saline
SLE: systemic lupus erythematosus
SLEDAI: SLE disease activity index
SLICC: Systemic Lupus International Collaborating Clinics
